# Antibody-associated epilepsies: Clinical features, evidence for immunotherapies and future research questions

**DOI:** 10.1016/j.seizure.2016.07.002

**Published:** 2016-10

**Authors:** Ochuko D. Bakpa, Markus Reuber, Sarosh R. Irani

**Affiliations:** a Academic Neurology Unit, Royal Hallamshire Hospital, University of Sheffield, Sheffield S10 2JF, UK; b Nuffield Department of Clinical Neurosciences, University of Oxford, Oxford OX3 9DS, UK

**Keywords:** Autoimmune, Autoantibodies, LGI1, NMDA receptor, GABA receptor, Immunotherapy, Faciobrachial dystonic seizures

## Abstract

**Purpose:**

The growing recognition of epilepsies and encephalopathies associated with autoantibodies against surface neuronal proteins (LGI1, NMDAR, CASPR2, GABA_B_R, and AMPAR) means that epileptologists are increasingly asking questions about mechanisms of antibody-mediated epileptogenesis, and about the use of immunotherapies. This review summarizes clinical and paraclinical observations related to autoimmune epilepsies, examines the current evidence for the effectiveness of immunotherapy, and makes epilepsy-specific recommendations for future research.

**Method:**

Systematic literature search with summary and review of the identified publications. Studies describing the clinical characteristics of autoantibody-associated epilepsies and treatments are detailed in tables.

**Results:**

Literature describing the clinical manifestations and treatment of autoimmune epilepsies associated with neuronal cell-surface autoantibodies (NSAbs) is largely limited to retrospective case series. We systematically summarize the features of particular interest to epileptologists dividing patients into those with acute or subacute encephalopathies associated with epilepsy, and those with chronic epilepsy without encephalopathy. Available observational studies suggest that immunotherapies are effective in some clinical circumstances but outcome data collection methods require greater standardization.

**Conclusions:**

The clinical experience captured suggests that clusters of clinical features associate well with specific NSAbs. Intensive and early immunotherapy is indicated when patients present with autoantibody-associated encephalopathies. It remains unclear how patients with chronic epilepsy and the same autoantibodies should be assessed and treated. Tables in this paper provide a comprehensive resource for systematic descriptions of both clinical features and treatments, and highlight limitations of current studies.

## Highlights

⿢ The field of autoimmune epilepsies has increasing relevance to general epileptology.
⿢ Established clinical features often associate closely with autoantibody specificities.
⿢ The diseases often respond well to early immunotherapies plus rapid escalation.
⿢ Randomized controlled trials are eagerly awaited.

## 1 Introduction

It has long been recognized that patients with autoantibody-associated systemic disorders (especially Systemic Lupus Erythematosus and Sjögren's Syndrome) have an increased risk of epilepsy [1]. In these conditions, the link with epilepsy is likely to be multifactorial and not solely related to direct actions of antibodies on neurons. Over the last decade, however, it has become clear that there are also a number of autoimmune conditions which present with epilepsy plus other neuropsychiatric manifestations, that are characterized by specific autoantibodies directed against neuronal targets. The study of these autoantibodies is especially informative as their specific molecular targets are likely to inform the mechanisms of epileptogenesis in humans. This review focuses on these emerging conditions as they now feature in the differential diagnosis of many other neurological and psychiatric disorders, especially those associated with epilepsy (Table 1). The targets of the major epilepsy-associated antibodies include the extracellular domains of neuronal proteins such as leucine-rich glioma inactivated-1 (LGI1), contactin-associated protein like 2 (CASPR2), the N-methyl-D-aspartate receptor (NMDAR) and the gamma aminobutyric acid receptor, plus intracellular molecules such as glutamic acid decarboxylase (GAD) [2], [3], [4], [5], [6], [7].

Neuronal cell-surface antibodies (NSAbs) are detected using various techniques such as immunohistochemistry, radioimmunoassays and cell-based assays [8]. Some NSAbs have not only been associated with epilepsy but also with other neurological manifestations involving the peripheral nervous system (for example neuromyotonia) or the central nervous system (for example psychosis and amnesia) [8], [9], [10], [11], [12]. The likely pathogenic role of these NSAbs is suggested by observations such as the temporal association between clinical improvement and reductions in antibody levels, the presence of these antibodies in cerebrospinal fluid (CSF), and early in vitro and in vivo animal experiments which use the human antibodies to reproduce aspects of the human disease. Increasing recognition of these autoantibody-mediated disorders may require some novel approaches to fundamentals of epilepsy including:

1. a partial reclassification of the aetiologies of epilepsies [13] with a focus on the diagnosis and definition of antibody-mediated epilepsies
2. the need to identify which patients with refractory or new-onset epilepsies may benefit from autoantibody testing, and
3. a more detailed understanding of the potential role for immunotherapy (IT) in the management of antibody-associated epilepsies.

We approach these issues by combining a systematic literature search with a review of the findings, and detailed reference summary tables. Our main focus is the use of ITs in the management of autoantibody-associated epilepsies. However, before addressing these treatment-related questions, we summarize current knowledge about the typical presentations of autoimmune encephalitis, with a focus on the epileptic phenotypes seen in these disorders and the range of associated neuropsychiatric manifestations. Subsequently, we discuss the significance of autoantibodies in patients with seizures in different clinical settings. In this way, our review also aims to improve recognition and diagnosis of these potentially treatable syndromes.

## 2 Methods

We conducted a systematic search of PUBMED for articles published between January 1995 to April 2015 using the search terms ⿿antibodies and epilepsy, VGKC and epilepsy, NMDA and epilepsy, AMPA and epilepsy, GAD and epilepsy⿿. The search was limited to articles in English. The list of identified articles was complemented by additional searches for relevant articles in the reference section of the publications captured by the initial search. References relating to IgA or IgM antibodies have not been included.

## 3 Results

567 studies were identified of which 34 were most relevant to this review (Table 2). The studies identified were observational studies and case reports. No controlled clinical trials were identified. The studies included describe a total of 1451 patients (182 male, 767 female, 502 gender not specified) with clinical presentations of antibody-associated epilepsies. Patient ages ranged from 2 to 92 years (median age 23). Most case series or patient groups were small, but ranged from 3 to 577 cases. Most patients described had a concomitant neuropsychiatric syndrome consistent with an encephalopathy.

## 4 Clinical characteristics of autoimmune epilepsies

The autoimmune encephalopathies associated with seizures and antibodies to LGI1, GAD and the NMDA-, AMPA-, and GABA_B_-receptors are summarized in Table 3, and described in more detail below with a particular focus on the aspects most relevant to epileptology.

### 4.1 Syndromes associated with LGI1-, CASPR2- and VGKC-complex-antibodies

The clinical features, radiological and CSF findings of patients with these antibodies are summarized in Table 2a. The nomenclature of these antibodies in the literature appears complex and is summarized below.

Since 2010, it has become apparent that ⿿VGKC-complex⿿ antibodies principally target the extracellular domains of LGI1, and less frequently CASPR2 [14]. Patients with antibodies against these two components of the VGKC-complex may be differentiated by some clinical features. For example, the presence of faciobrachial dystonic seizures (FBDS) and hyponatraemia strongly suggest LGI1-reactivity, whereas the presence of neuromyotonia or other features of Morvan's syndrome suggest CASPR2-directed autoantibodies (sometimes accompanied by LGI1-antibodies). In other cases, the antigenic target is difficult to predict on clinical grounds. By comparison to VGKC-complex antibody determination, the detection of LGI1 or CASPR2-reactivity offers very good specificity for an antibody-mediated neurological syndrome, whereas low-titre VGKC-complex antibodies (between 100 and 400 pM) were noted in an elderly stroke cohort at a frequency of around 6%, rates of LGI1- and CASPR2-antibodies are much lower in controls [7], [14], [15], [16], [17], [18]. Therefore, we preferentially use the terms LGI1- and CASPR2-antibodies below. The remaining VGKC-complex antibodies without LGI1 and CASPR2 reactivities are termed ⿿double-negative⿿ and their clinical significance is less clear. We reserve the term ⿿VGKC-complex antibodies⿿ when LGI1 and CASPR2-antibody assays were not performed (often prior to 2010).

Patients with LGI1-antibodies are often males (65%), older than 50 years, and present with features of acute or subacute amnesia, confusion, sleep disturbances, and both frequent FBDS and medial temporal lobe seizures. In under 10% of cases, the condition is found to be paraneoplastic, usually associated with thymoma, or more rarely small cell lung cancer (SCLC) [7], [14], [19]. In around 60% of cases with LGI1-antibodies, unilateral/bilateral hippocampal high signal is found in the mesial temporal lobe on magnetic resonance imaging (MRI), consistent with limbic encephalitis (LE) [7], [14], [20]. This is usually localized to the hippocampus and amygdala, occasionally straying into the insula cortex or parahippocampal gyrus. In addition, there have been more recent reports of basal ganglia and hypothalamic involvement in MRI studies of such patients [21], [22], [23]. However, around 40% of patients show no imaging abnormalities and therefore clinical recognition remains paramount. The VGKC-complex/LGI1-antibody serum concentration is raised compared to that in the CSF at disease onset [7], [14], [20] and in a few cases with well-defined syndromes (such as FBDS) LGI1-antibodies are undetectable in CSF (Irani, unpublished observations) [24]. Typical encephalitic CSF findings, such as lymphocytic pleocytosis, oligoclonal bands (OCBs) and elevated protein, are uncommon: routine CSF analysis is normal in around 60% of patients [7], [20]. Other characteristic findings include serum hyponatremia, often caused by syndrome of inappropriate ADH secretion (SIADH) [7], and abnormal electroencephalogram (EEG) appearances with temporal spike waves and frontotemporal slowing [14], [25], [26].

Over 70% of seizures reported in studies were of focal onset with secondary generalization [7], [19], [23], [27], [28], however in some generalized tonic-clonic seizures a focal onset was not apparent [7], [28], [29], [30]. The characteristic semiology termed FBDS has been described in patients with LGI1-antibodies and will be discussed below [22], [31]. In addition, seizures with prominent bradycardia and piloerection appear more suggestive of a LGI1-antibody-associated syndrome [32], [33].

More unexpected manifestations have also been reported with these antibodies. For example, VGKC-complex antibodies (often CASPR2-antibodies) have been associated with chronic, often analgesia-refractory, pain. However, only 2% of patients in this study had seizures, suggesting some non-epileptogenic phenotypes are associated with these antibodies [34]. This is also true for others patients with double-negative VGKC-complex antibodies [14], [16], [18]. Indeed, perhaps consistent with a lack of seizures in this cohort, the pathogenic relevance of double-negative antibodies remains uncertain [11], [35]. Therefore, LGI1- and CASPR2-antibodies are of much greater relevance to epileptologists.

### 4.2 Faciobrachial dystonic seizures (FBDS)

FBDS represent a distinctive seizure semiology which were first reported in three patients [31] and subsequently the term was coined after observation of 26 additional cases [22]. Following these initial descriptions, multiple cases have been recognized and reported worldwide [26], [36], [37], [38], [39], [40]. FBDS are characterized by brief episodes (usually <3 s) of synchronous dystonic arm posturing and ipsilateral facial grimacing occurring at a median of 50 times per day. Some patients can also develop longer episodes and prominent leg involvement. A clinically important link between FBDS and development of cognitive impairment (CI) has been observed: around 70% of patients developed CI a median of 35 days following the onset of FBDS, while another 20% developed FBDS after the onset of CI. Interestingly, around 10% of patients with FBDS, who received IT and antiepileptic drugs (AEDs), never progressed to develop CI [22]. In a prospective study of ten patients with FBDS, eight of ten patients who received AEDs or no treatment developed CI. By contrast, the two patients who received IT did not develop CI [41]. Overall, AEDs were only effective in about 10% of patients, and frequently resulted in severe cutaneous reactions. By contrast, FBDS typically respond to IT with corticosteroids and sometimes plasma exchange (PLEX) [22], [41]. In another study involving 14 patients with LGI1-antibodies, at 1 month follow-up a superior response was observed in patients treated with corticosteroids and IVIG, in contrast to those who only received corticosteroid treatment [26].

### 4.3 NMDAR-antibody associated encephalitis

NMDAR-antibody associated encephalitis is a recently described disorder in which infrequent seizures are associated with the presence of autoantibodies directed against the extracellular domain of the NR1 subunit of the NMDAR. Table 2b focuses on the clinical and electrographic-features of relevant studies. NMDAR-antibody associated encephalitis is associated with a characteristic set of clinical features which were first reported in 12 young women who all had a diffuse encephalopathy with seizures and psychiatric symptoms in association with teratomas (ovarian *n* = 11, mediastinal *n* = 1) [2]. Importantly, patients showed significant improvements following tumour removal and IT. Subsequently, it has been recognized that the disease can affect both males and females, and that it is non-paraneoplastic in around 70% of cases, especially younger children [3], [42], [43], [44], [45], [46].

The clinical features associated with NMDAR-antibodies often evolve in stages [3], [47], [48]. The syndrome typically begins with prodromal low-grade fever, with headache and fatigue unrelated to a known infectious aetiology. The subsequent phase is characterized by delusions, hallucinations (visual or auditory) and personality change in addition to speech difficulties, disorientation and seizures. In a study of 100 patients, seizures were reported in 76, with generalized tonic clonic seizures (GTCS; *n* = 45), complex partial seizures (CPS; *n* = 10), secondary generalized seizures (*n* = 8), refractory status epilepticus (*n* = 6), focal motor seizures (*n* = 7) and epilepsia partialis continua (*n* = 2) [43]. Following a lag of 10⿿20 days, patients typically progress to a phase with abnormal movements (such as orofacial dyskinesia and choreoathetoid movements), reduction in consciousness and florid dysautonomia, often necessitating intensive care admission [3], [48]. Although the characteristic movements of the hyperkinetic phase are carefully described [49], they have been confused with seizures [48] despite scalp EEGs usually demonstrating no seizure activity. However, both clinical and electrographically-determined seizures and a movement disorder can sequentially appear in a single limb within an individual patient over seconds, suggesting a set of common neuronal networks which may be differentially engaged to generate the relative cortical versus subcortical activity [50].

In addition, NMDAR-antibodies have been identified in patients with new-onset epilepsies. Niehusmann and colleagues reported NMDAR-antibodies in 5 of 19 women (age range 15⿿45 years). All patients had extratemporal epilepsy which was often, but not always, associated with neuropsychiatric symptoms, reduced level of consciousness and speech disturbance. Other features included nystagmus, dyskinesia, dystonia and hypoventilation. All patients were started on AEDs in the first 2 months of seizure onset. At median follow up of 26 months, seizure relapse was seen in two patients on AEDs. Clinical improvements in seizure frequency were seen in three patients treated with IT comprising of corticosteroids and IVIG.

Imaging has a limited role in a positive diagnosis of NMDAR-antibody encephalitis as MRI and CT findings are often normal or non-specific, despite the presence of multifocal neurological dysfunction [3], [43]. Indeed, non-specific changes involving the medial temporal lobe, basal ganglia, periventricular or subcortical areas, pons and cerebellar cortex may be seen in about 20% of cases [2], [51], [52]. However, recent studies indicate abnormal resting state and diffusion tensor imaging [53], and that NMDAR-antibody encephalitis may occur simultaneously with clinical and MRI features suggestive of demyelinating disorders [54], [55]. A distinctive EEG finding in NMDAR-antibody encephalitis is a generalized slow delta wave with a rhythmic fast beta wave; this pattern is described as the extreme delta brush because of its similarity with waveforms seen in premature infants [56].

The serum and CSF should both be examined because while NMDAR-antibody titres are consistently around 10 times higher in the serum than CSF, the lower total immunoglobulin G (IgG) concentration in CSF appears to offer a better signal to noise ratio in the immunoflourescent assay [3], [57], [58]. Also, CSF shows early lymphocytic pleocytosis in 80% of cases, and a later presence of OCBs (60% of cases) [3], [43].

Other than the presence of a NMDAR-expressing ovarian tumour, likely to be an immunizing factor, causes of NMDAR-antibody generation have remained obscure. However, recent studies have demonstrated the association of herpes simplex virus encephalitis (HSVE) with NMDAR-antibodies [9], [59], [60]. In a retrospective study of patients with HSVE, serum and CSF NMDAR-antibodies were detected in 13 of 44 cases both early and late after HSVE [60]. In addition, days to weeks after HSVE, relapses of encephalopathy with choreoathetosis have been associated with de novo generation of NMDAR-antibodies, and provide another mechanism for NMDAR-antibody generation [9], [59]. Other recent studies have shown Creutzfeldt-Jakob disease [61] and Varicella-Zoster virus encephalitis as potential triggers of NMDAR-antibodies [62], and collectively suggest a group of illnesses which may trigger antibody-mediated epilepsies.

### 4.4 GAD-antibody associated encephalitis

Glutamic acid decarboxylase (GAD) is an intracellular enzyme that catalyses the conversion of L-glutamic acid to gamma-aminobutyric acid (GABA). GAD is expressed in GABAergic neurons and pancreatic β-cells. Antibodies to GAD are associated with several autoimmune disorders including type 1 diabetes mellitus [63], Stiff Person Syndrome (SPS) [64], and cerebellar ataxia [65]. Recently, high titres of GAD-antibodies were recognized in a subgroup of patients with non-paraneoplastic LE (Table 2c). In a study involving 53 patients with LE, nine had elevated levels of GAD-antibodies of which seven were females who presented predominantly with temporal lobe seizures. The epileptic seizures were refractory to both AEDs and IT, by contrast to seizures associated with LGI1-antibodies [66]. MRI findings in most studies show unilateral or bilateral mesiotemporal high signal on T2/FLAIR, consistent with the characteristic presentation of memory disturbance and temporal lobe epilepsy seen in these patients [67]. This appearance, as with other causes of LE, may evolve to atrophy and hippocampal sclerosis. The CSF analysis typically shows the presence of OCBs, lymphocytic pleocytosis, elevated protein and intrathecal synthesis of GAD-antibodies [68]. Recently, GAD-antibodies, especially in elderly male patients, have been shown to more commonly suggest an increased risk of an underlying tumour [69] and as a non-paraneoplastic syndrome in children [70].

### 4.5 Gamma aminobutyric acid (B) receptor (GABA_B_R) antibody associated encephalitis

GABA_B_Rs are critical inhibitory neuronal receptors. Antibody-mediated disruption of these receptors has been linked with seizures and changes in memory and behaviour (studies summarized in Table 2d). In an observational study involving 15 patients with GABA_B_R-antibodies and LE (median age 62 years, range 24⿿75 years), all presented with seizures, confusion and memory dysfunction. Seizures were the predominant presenting feature in 13, and were mostly of temporal lobe onset with secondary generalization. Two patients presented with status epilepticus. Seven patients had tumours, of which five had SCLC. CSF findings showed lymphocytic pleocytosis (*n* = 4) and MRI showed increased signal, typical of LE. Clinical improvement was seen in six patients who received IT alone and three who had IT and tumour removal. There was no clinical improvement in four patients without IT [5].

In another study, GABA_B_ receptor-antibodies were identified in ten patients with similar demographics, prominent seizures and frequent SCLC. Only two patients made a complete recovery, four had partial responses and three had no response [71]. Separately, antibodies to GABA_B_ receptors were identified in 10 patients (median age 70 years); five had SCLC. MRI showed bilateral mediotemporal (*n* = 9) and cortical (*n* = 2) abnormalities. EEG demonstrated encephalopathy, partly with epileptiform discharges. Five patients received IT, two had tumour treatment, and three both therapies. One patient recovered fully, one improved slightly, five experienced cognitive decline and three died [72]. Another study confirmed many of these findings and added ataxia, opsoclonus and status epilepticus to the LE syndrome [73].

### 4.6 α-Amino-3-hydroxy-5-methyl-4-isoxazolepropionic acid receptor (AMPAR)-antibody associated encephalitis

Antibodies to the AMPAR have recently been described in patients with LE. Not only are AMPAR-antibodies the least frequent of these antibodies (Waters and Irani, unpublished) but Table 2e suggests they are also the least epileptogenic. In an observational study involving ten patients with AMPAR-antibodies (age range 38⿿87, median age 60) and LE who presented with confusion, amnesia and disorientation, seven patients had tumours (lung (*n* = 2) breast (*n* = 2) and thymus (*n* = 3)) and only four patients had focal seizures (*n* = 2) or GTCS (*n* = 2). Nine patients were treated with IT (IVIG, corticosteroids, PLEX and azathioprine), and six patients underwent tumour resection. All nine patients had good responses to IT and five patients had relapses at 2⿿101 months after the initial episode of encephalitis [4]. In other studies, AMPAR-antibodies were detected in three patients presenting with amnestic disturbances [72], and in others presenting with psychosis [74]. Recent series prove this disorder is commonly paraneoplastic [75], [76].

## 5 Autoantibodies in patients with chronic refractory epilepsy

The autoantibodies associated with autoimmune encephalopathies, discussed above, have also been detected in unselected patients with recent onset and chronic forms of isolated epilepsies. McKnight et al. reported the presence of VGKC-complex and GAD-antibodies in 67 patients presenting with drug resistant epilepsy [28]. The group with VGKC-complex antibodies was derived from a cohort selected due to a high clinical suspicion of autoimmunity, and consisted of 16 patients; eight presented with a short duration (1⿿16 weeks) of seizures. Patients with a short duration of epilepsy often had higher titres of VGKC-complex antibodies and five of these six patients were treated with IT (oral corticosteroids, PLEX and IVIG) and made a good recovery. In contrast, all five GAD-antibody positive patients were females with long durations of refractory epilepsy with onset in childhood or early teens, and a poorer IT response. In a subsequent study involving 106 patients (all females) with chronic refractory epilepsy, voltage gated calcium channel (VGCC) antibodies (>45 pM) were slightly raised in one patient, GAD antibodies were absent (<3 U/ml) in all patients, and six patients had raised VGKC-complex antibody titres (118⿿1406 pM, normal range <100 pM). Seizures were mainly GTCS, tonic or atonic with childhood onset, and MRIs were normal in the six patients with VGKC-complex antibodies [77]. In a retrospective study of patients with refractory epilepsy and a suspected autoimmune aetiology, 18 of 32 had VGKC-complex antibodies, seven had GAD65-antibodies, and one patient had NMDAR-antibodies. Other antibodies identified included collapsin response mediator protein-5 (CRMP-5; *n* = 2), Ma2 (*n* = 1), and ganglionic acetylcholine receptors (*n* = 1). In this study, 81% of patients improved clinically after IT, with 67% achieving seizure freedom within a period of 10 months [29]. Overall, the patients with autoantibodies often had frequent, AED-refractory seizures with neuropsychiatric comorbidities. Interestingly, these features overlap with the autoimmune epilepsies described above, and suggest common themes in the phenotype of autoimmune epilepsies, and perhaps a rationale to select patients for antibody testing.

In another study of 29 patients with autoimmune epilepsy, 18 responded positively to a trial of IT (iv methylprednisolone 1,000 mg or IVIG 0.4 g/kg daily for 3⿿5 days, followed by once weekly infusions of iv methylprednisolone or IVIG for 6⿿12 weeks at the same dose). Ten patients became seizure free, the others had more than 50% reduction in seizures following treatment. 43% of patients who failed to respond to first-line agents (iv methylprednisolone or IVIG) improved following treatment with second-line agents such as rituximab [78]. Therefore, several studies suggest the presence of autoantibodies with pathogenic potential in the serum of patients with epilepsies. In those patients with a more abrupt onset of seizures, multiple studies provide consistent observational evidence to suggest a preferential response to IT over AEDs.

However, the results of these studies cannot yet readily be generalized to the wider population of patients with epilepsy for several reasons. Firstly, none of these studies have consistently measured the presence of autoantibodies in the CSF. This has been a requisite of some studies [57] and intuitively suggests a greater likelihood of pathogenicity. However, it is not known whether serum NSAbs alone have sufficient CNS-pathogenicity without accompanying detectable CSF antibodies, nor is the pathogenic necessity of intrathecal antibody synthesis well understood. Animal models suggest that antibodies can cross the blood brain barrier, and we know some patients with classical FBDS and serum LGI1-antibodies do not have CSF LGI1-antibodies. In these patients, there is little doubt that such a distinctive clinical syndrome is related to the presence of LGI1-antibodies which are detectable in serum only. By extension, the presence of serum LGI1 or CASPR2-antibodies in unselected patients with epilepsy of unknown aetiology may be sufficient evidence to prompt ITs, and in two studies this approach was employed with some benefits [79], [80]. While the relative roles of CSF and serum remain incompletely understood, we recommend testing of both CSF and serum in all patients when possible, as these cumulative data will give clinicians the best opportunity to understand and monitor the patient's disease. Secondly, no study has prospectively evaluated the frequency of autoantibodies in unselected patients with a first adult-onset seizure to determine their wider relevance. An increased autoantibody frequency in patients with acute versus more chronic forms of epilepsy, would perhaps suggest the acute onset cases derive the most benefit from IT. Thirdly, sequential results of seizure frequency and antibody-levels need to be determined in several individual patients over time. Such correlations can be very close in patients with LGI1-antibodies and FBDS, but seizures can also often stop before antibody levels fall markedly [22], [41], [80]. Finally, it is currently unclear whether a substantial number of epilepsy patients without encephalopathic features respond well to ITs. Certainly, patients with FBDS usually do. However, other cohorts demonstrating a good IT response included patients with frequent MRI and cognitive changes, suggesting these patients may have been classified with an autoimmune encephalopathy rather than a pure antibody-associated epilepsy [78].

All of these questions return to the important issue of ITs. Of course, an IT response is of great potential interest to epileptologists, as around 30% of patients with epilepsy are currently AED-refractory. Also, paradigms already exist for efficacy of ITs in epilepsies: for example, corticosteroids and PLEX are advocated in the treatment of refractory status epilepticus [79], [80], [81]. However, to be certain of efficacy, a randomized clinical trial is required. In the interim, immunosuppressive therapy could be reserved for patients with NSAbs, and either AED-refractory seizures or explosive onset of seizures, particularly in the context of associated neuropsychiatric features.

## 6 Treatment and outcomes in antibody-associated epilepsies

**Fig. 1:**
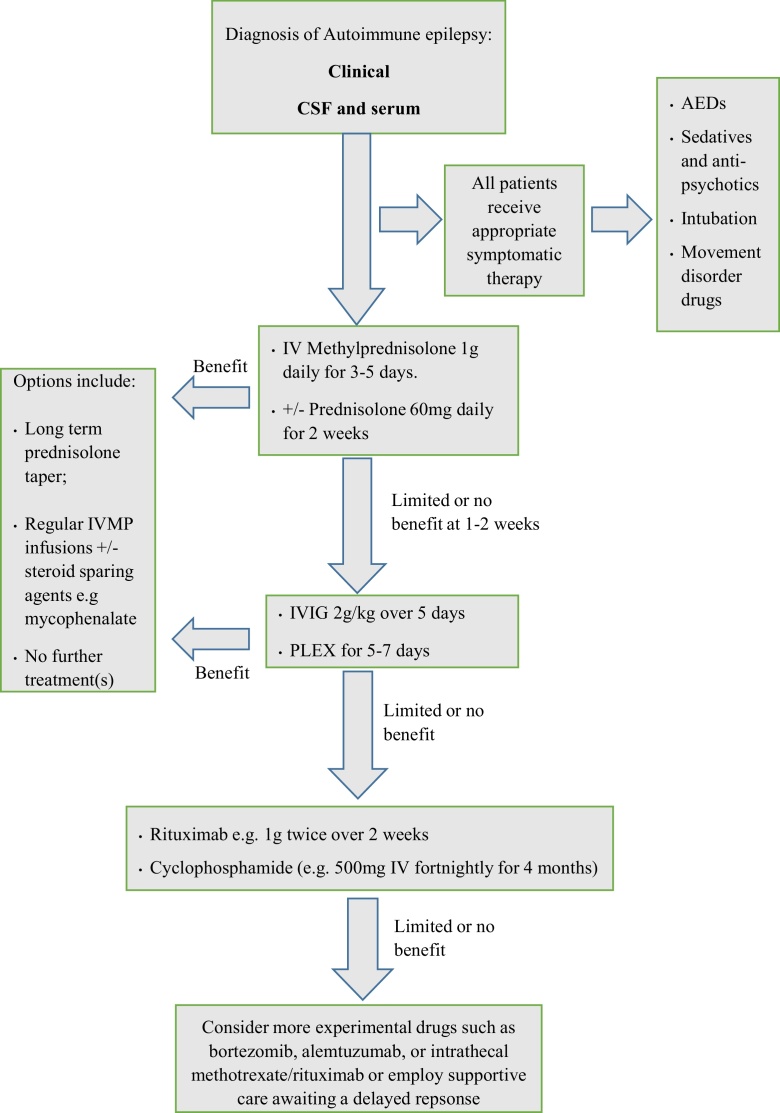
Immunotherapy escalation options in acute onset/encephalopathic autoimmune epilepsy. The time period to wait before determining whether treatment is successful, or of limited or no benefit, is unclear. Likewise, there are currently no agreed criteria on what degree of improvement is deemed satisfactory benefit.

The striking differences in the clinical presentation between the various NSAb-associated epilepsies suggest that a ⿿one size fits all⿿ approach to treatment would not be appropriate. Treatments must be tailored to the individual. The paragraphs below summarize the most commonly pursued treatment strategies, and available observational evidence. There are no standard protocols regarding the particular ITs which are most appropriate for the different forms of NSAb-mediated disorders. Fig. 1 summarizes a proposed treatment regimen for patients with encephalopathic or acute onset of autoimmune epilepsy. Certainly, patients with CSF and serum autoantibodies who are severely ill should be given rapid escalation of ITs. Similarly, patients with a syndrome that is consistent with an autoimmune aetiology, regardless of autoantibody status, are also obvious candidates for ITs. The debate is less clear-cut around patients with few features of autoimmune epilepsy (such as infrequent, typical temporal lobe seizures plus no neuropsychiatric features) and serum-only autoantibodies. In these patients, a staged approach seems reasonable, whereby perhaps only AED-refractory patients receive a predefined course of ITs with accurate baseline and post-treatment measurements of seizure frequency. However, the administration of ITs ⿿ in terms of number of therapies, doses and durations ⿿ often depends on the antibody in question. Therefore, the IT regimes discussed below are considered in the context of the antigenic target. Importantly, assuming limited reporting bias, Table 2 suggests a low rate of side-effects from ITs, not different to AED-induced side effects.

### 6.1 LGI1- and CASPR2-antibody associated encephalopathy

Studies describing the treatment of LGI1-antibody associated encephalopathy are summarized in Supplementary Table 1A. Some observational studies have provided evidence for high-dose steroids, IVIG and PLEX for patients with autoantibodies to LGI1 and CASPR2 [7], [11], [26], [82]. In a retrospective study of ten patients with high titres of VGKC-complex antibodies who presented with seizures and memory disturbance, IT (including IVIG 2 g/kg/day, 100 mg prednisolone on alternate days and PLEX for 5 days) resulted in significant clinical improvement in frequency of seizures and cognition in six patients within 2 weeks⿿12 months, correlating with reductions in antibody titres [7]. Earlier ITs, and possibly corticosteroids, appeared to provide greater benefits.

In another retrospective study of nine patients with VGKC-complex antibodies and LE, the IT regimen consisted of PLEX (50 ml/kg), IVIG (2 g/kg) and iv methylprednisolone (1 g thrice) followed by a maintenance dose of oral prednisolone (starting at 1 mg/kg/day). Significant clinical improvements were observed, as seizures stopped in all nine patients within 1 week of treatment and cognitive functions improved in all patients within 3 months. VGKC-complex antibody titres returned to normal within 1⿿4 months [82]. In agreement with these seizure-related observations, despite a typically poor response to AEDs, FBDS are often exquisitely sensitive to corticosteroids and PLEX. A small prospective study has suggested termination of FBDS may be achieved very rapidly (often within days ⿿ few weeks) with these agents, and that cessation of FBDS may be associated with a prevention of cognitive impairment [41].

Of 14 patients with LGI1-antibodies (median age 60.5 years, range 41⿿78), all presented with seizures (ten with FBDS, four with status epilepticus) and twelve showed changes in memory and behaviour. Seven patients were treated with corticosteroids only: all seven showed clinical improvement. Further improvements were seen following administration of IVIG. Five patients received combined IT (corticosteroid and IVIG) immediately after diagnosis: four of these achieved seizure freedom without relapse. The five patients in this case series who received IT within 1 month of symptom onset showed the greatest improvement in seizure frequencies [26].

IT with rituximab has been reported to have beneficial effects in only few patients with LGI1-antibodies. Only one of seven patients who received rituximab showed a clear clinical improvement, and benefits were more equivocal in three others [39], [83]. However, while patients with LGI1-antibodies appear to improve markedly in the shorter-term with IT [7], [14], [34], the modified Rankin Scale outcomes at 4 years do not appear to differ between groups treated with steroids plus IVIG and/or PLEX, or steroids alone [11]. Furthermore, these retrospective observational findings should be taken in the context of a few reports showing similar improvements in patients without ITs [84]. This is in concordance with the observation that most of these patients relapse infrequently and make good improvements with steroids alone.

### 6.2 NMDAR-antibody associated encephalitis

Patients with NMDAR-antibody associated encephalitis often pose significant therapeutic challenges, principally related to the long natural history of the disease and its tendency to frequent relapses [3], [43]. The available treatment data for NMDAR-antibody associated encephalitis are summarized in Supplementary Table 1B. In a study involving 100 patients, 92 received ITs with corticosteroids (*n* = 76), IVIG (*n* = 62) or PLEX (*n* = 34) and 51 patients underwent tumour resection. At median follow up of 17 months (range 1⿿194), 47 had made a full recovery, 28 had a mild stable deficit, 18 had severe deficits and 7 died. At a median of 18 months, relapses were seen in 15 of the 100 patients [43]. In another study, patients with non-paraneoplastic NMDAR-antibody encephalitis recovered faster if commenced on IT less than 40 days after symptoms onset [3], and relapses were noted in 10 of 35 (29%) patients who often received a short duration of ITs [3]. In the largest observational study of 577 patients, 472 patients (94%) received first line IT (IVIG, PLEX and steroids) or tumour removal (found in 38%), with 53% experiencing clinical improvements within 4 weeks. In the group of 221 patients without clinical benefits from first-line IT, second-line IT (rituximab and cyclophosphamide) was instituted in 57% with better clinical outcomes reported in these patients compared to those who did not receive second line treatments. Those receiving second line ITs also had fewer relapses [45]. In summary, observational data suggest early and escalating ITs show efficacy in patients with NMDAR-antibody encephalitis.

### 6.3 GAD-antibody associated encephalitis

GAD-antibody associated disorders often show a disappointing clinical response to ITs despite aggressive approaches and simultaneous use of AEDs. In an observational study involving patients with LE and GAD-antibodies (*n* = 9) or VGKC-complex antibodies (*n* = 10), there was a marked improvement in seizure frequency in the latter group. But in the GAD-antibody group, no patient became seizure free. GAD-antibodies, unlike those against LGI1, remained elevated during treatments [66], and, of course, target an intracellular antigen suggesting a lack of direct pathogenicity. Accordingly, a case series of 112 patients with unexplained adult onset epilepsy, showed GAD-antibodies were found in six (5.4%) of which five received IT without seizure freedom [68]. Supplementary Table 1C summarizes studies describing the treatment of GAD-antibody associated encephalitis.

## 7 Discussion

In recent years, there has been accumulating evidence to support an autoimmune aetiology for some patients with AED-resistant seizures, typically in the context of an antibody-mediated encephalopathy [22], [28], [37]. The molecular precision of the likely pathogenic autoantibody implies that the study of any seizures generated by these autoantibodies ⿿ not just those which are clinically distinctive ⿿ may inform mechanisms of epileptogenesis, and the therapeutic options. Therefore, any seizure disorder likely to be caused by these antibodies is an example of an ⿿autoimmune epilepsy⿿. Yet it remains uncertain how these autoimmune epilepsies should be treated. Consistently, early ITs, and sometimes more aggressive regimes, produce better outcomes [3], [11], [26], [43], [45]. However, to date, no randomized interventional studies have been conducted. Such trials will be challenging to implement for many reasons including the heterogeneous clinical presentation of cases [29], the understandable reluctance of clinicians to abstain from immediate and empirical treatments, the absence of proven high-quality outcome measures other than seizure frequency, and the sometimes considerable time lag between onset of symptoms and diagnosis [2], [7], [8], [25], [48]. In the absence of RCTs, high-quality observational studies characterized by standardized and complete assessments of clinical outcomes are of value. However, evidence from observational studies is not without limitations. These include concomitant under-reported use of varied AEDs [29], the absence of a placebo-treated group or natural history cohort, small numbers in many studies, and the likely publication bias from reporting of marked recoveries. Another clear observation from this review is that patients with autoimmune epilepsies are inconsistently phenotyped [3], [45], [52], [85]. For example, seizure semiologies were only rarely described in detail. More importantly, outcome measures were inconsistent, and while the administered ITs were described in all papers, the details of AED administration were markedly under-reported. For example, only 3 of the 11 (27%) VGKC-complex antibody studies reviewed described whether AEDs were used before IT, only 5 of 11 (45%) reported the number of AEDs administered, and only 4 of 11 (36%) showed seizure frequency as an outcome measure. For NMDAR-antibodies, the corresponding percentages were 0%, 40% and 0%. Epilepsy-specific data must be more consistently reported in future studies and a minimal dataset might include longitudinal measures of mRS, seizure frequency, autoantibodies plus use of IT and AEDs. This may create the basis for the definition of IT-responsive antibody-associated epilepsies.

Although there is accumulating evidence for the presence of antibodies in patients with refractory epilepsy [3], [6], [27], [28], [29], [82], there are several unanswered questions such as the pathogenic role of these antibodies, the optimal methods used to identify patients with epilepsy and autoantibodies, and there are only paradigms for a clear IT-response in patients with more acute encephalopathic syndromes [29]. With the exception of GAD-antibody associated disorders, all other autoimmune conditions described in this review are characterized by NSAbs: antibodies that target extracellular domains of neuronal proteins located on the cell membrane. The antibodies may therefore be capable of exerting their pathological effects in the brain, but how these antibodies gain access to the central nervous system, the mechanisms by which they cause changes in the neuronal networks and why they exert their pathology in specific regions of the brain remains to be elucidated. Furthermore, whether the marked intrathecal synthesis of antibodies in GAD [86] and NMDAR-antibody [87] associated disorders drives the pathogenic process requires further investigation.

The selection of patients for autoimmune evaluation requires a high level of suspicion at initial consultation. In view of the fact that there are currently no universally agreed diagnostic criteria for autoimmune epilepsies, it is difficult to identify patients who will benefit from antibody screening. However, evidence from observational studies suggests that high frequency of seizures, psychiatric comorbidity and resistance to AEDs are useful indicators. In conclusion, despite the lack of definitive RCTs, this review provides evidence to suggest that early, often multiple, ITs used in a graded fashion (see Fig. 1) are efficacious and safe in patients with autoimmune epilepsy.

## Role of the funding source

SRI is supported by the Wellcome Trust Intermediate Clinical fellowship, the British Medical Association ⿿ Vera Down Research Grant and is part of the Oxford Epilepsy Research Group.

## Conflict of interest statement

SRI is a coapplicant and receives royalties on patent application WO/2010/046716 entitled ⿿Neurological Autoimmune Disorders⿿. The patent has been licensed to Euroimmun AG for the development of assays for LGI1 and other VGKC-complex antibodies.

## Figures and Tables

**Table 1:** Disorders which may mimic autoimmune encephalopathies.

Differential diagnosis		Clinical features
Infections	Herpes simplex encephalitis	Fever, headache, personality change, seizures, memory loss, focal signs
	HIV	Seizures, psychosis, sleep disturbance, amnesia
	Enterovirus	Drowsiness, cerebellar ataxia, seizures
Psychiatric disorders	Depression	Poor concentration, irritability, insomnia, fatigue, suicidal ideations
	Schizophrenia	Hallucinations, delusions, speech problems and limited cognitive impairment
	Bipolar disorder	Irritability, insomnia, fatigue, poor concentration
Inflammation	Cerebral vasculitis	Headache, confusion, seizures, focal signs
	Hashimoto's encephalopathy	Psychosis, myoclonus, tremor, poor concentration, amnesia
Structural lesions	Brain tumour, for example	Headache, vomiting, seizures, focal signs
Toxic/metabolic encephalopathy	Electrolyte imbalances (e.g. renal/liver/glucose)	Seizures, confusion, weakness, coma, muscle cramps
	Drugs (e.g. ketamine/cocaine)	Insomnia, confusion, nausea and vomiting
	Wernicke-Korsakoff syndrome	Confusion, amnesia, confabulation, ophthalmoplegia, ataxia
Degenerative	Creutzfield Jakob or rapid forms of Lewy Body or Alzheimer's disease	Amnesia, sleep fragmentation, myoclonus, anxiety, depression, visual hallucinations, parkinsonism

**Table 2:** Summary of autoimmune epilepsy studies. Studies are derived from search criteria in methods and details have been included only when they were available in the article. MRI findings: increased T2/FLAIR signal in structures listed within the table otherwise stated and studies showing multiple regions of brain involvement are further described in Supplementary Table 2. AEDs = Antiepileptic drugs; CPS = Complex partial seizures; CLN = Cortical laminar necrosis; CI = Cognitive impairment; DN = Double negative VGKC-complex antibody (without LGI1 or CASPR2 reactivities); EPC = Epilepsia partialis continua; FBDS = Faciobrachial dystonic seizures; FS = Focal seizure; FS+ = Focal seizure with impaired awareness; FM = Figural memory; FU = Follow-up; FLAIR = Fluid attenuated inversion recovery; GTC = Generalized tonic clonic seizures; GS = Generalized seizures; GAD = Glutamic acid decarboxylase; GABA = Gamma amino butyric acid; IT = Immunotherapy; IGE = Idiopathic generalized epilepsy; IVIG = Intravenous immunoglobulins; IgG = Immunoglobulin G; MDZ = Midazolam; MMSE = Mini-mental state examination, MTL = Medial temporal lobe; MTS = Mediotemporal sclerosis; MRI = Magnetic resonance imaging; mRS = Modified rankin score; N/A = Data not available; OCB = Oligoclonal bands; PCPC = Paediatric cerebral performance category scale; PL = Pleocytosis; PLEX = Plasma exchange; PPF = Propofol; ⿿Prot = Protein elevation; SGTCS = Secondary generalized tonic clonic seizure; SPS = Simple partial seizures; SE = Status epilepticus; SIADH = Syndrome of inappropriate ADH secretion; SCLC = Small cell lung cancer; TLE = Temporal lobe epilepsy; TICS = Telephone interview of cognitive status (scored out of 41); VM = Verbal memory.

Author	Type of study/number of patients/antibody type	Clinical presentation	Types of seizure	EEG	MRI	CSF	AED given before IT? (Number/duration)	Number of AEDs used with IT	IT used/outcome	FU duration (range, median)/drug side effects
**a. LGI1/CASPR2/VGKC-complex antibody studies**										
Vincent et al., 2004	Observational *n* = 1 0 Female = 1 LGI1 (100%, retrospective)	Amnesia, Seizures, Confusion	GTC (*n* = 3), FS (*n* = 3), GTC + CPS/SPS (*n* = 3)	Normal (*n* = 2), Diffuse slowing + focal activity (*n* = 4), Focal activity (*n* = 2), Diffuse slowing (*n* = 1), Focal activity + diffuse slowing (*n* = 1)	Normal (*n* = 2), Unilateral MTL (*n* = 3), Bilateral MTL (*n* = 5)	OCB (*n* = 5), Mild PL (*n* = 5)	N/A	N/A	**IT:***n* = 10; steroids, PLEX, IVIG **Outcome:** Neuropsychology: marked recovery (*n* = 6), slight recovery (*n* = 3), no recovery (*n* = 1)	2 weeks⿿12 months/N/A
Thieben et al., 2004	Retrospective *n* = 7 Female = 2 VGKC-complex specificity unknown	Seizures, Amnesia, Irritability, Apathy	SPS (*n* = 2), CPS (*n* = 5), GTC (*n* = 2), SE (*n* = 1)	Epileptiform discharges (*n* = 4), Mild bitemporal slowing (*n* = 1), Diffuse/generalized slowing (*n* = 2)	Unilateral MTL (*n* = 1), Bilateral MTL (*n* = 6)	OCB (*n* = 1), ⿿Prot (*n* = 2)	N/A	N/A	**IT**: *n* = 6; steroids. One spontaneous recovery. **Outcome. TICS:** Good recovery (*n* = 3; TICS = 34⿿35/41), Partial recovery (*n* = 3; TICS = 27-33/41), Spontaneous (*n* = 1; TICS = 36/41))	7⿿36 months (median = 24)
Mcknight et al., 2005	Case control *n* = 16 Female = 9 VGKC-complex specificity unknown	Amnesia, Seizures, Depression	GTC (*n* = 9), CPS (*n* = 7)	N/A	Normal (*n* = 9), Hippocampal (*n* = 3), Claustrum (*n* = 1)	N/A	N/A	N/A	**IT:***n* = 6; steroids and IVIG. **Outcome:** Good response (*n* = 5), No response (*n* = 1)	2⿿60 months (median = 18)
Lai et al., 2010	Observational *n* = 57 Female = 2 0 LGI1 (100%) CASPR2 (2%)	Amnesia, Seizures	Temporal lobe seizures (*n* = 42), Myoclonus (*n* = 18)	Epileptiform discharge (*n* = 4), Diffuse or focal slowing (*n* = 11), Seizures (*n* = 11)	Temporal lobe (*n* = 43)	PL (*n* = 8), ⿿Prot (*n* = 13)	N/A	N/A	**IT**: *n* = 48; steroids, PLEX, IVIG. **Outcome:** Full recovery (*n* = 12), Moderate disability (*n* = 8), Mild disability (*n* = 27), Death (*n* = 3).	
Irani et al., 2011	Observationa *n* = 29 Female = 10 LGI1 (88%) CASPR2 (12%) Double negative (12%)	Amnesia, Confusion, Hallucinations, Depression, Dysautonomia, Seizures	FBDS	Normal (*n* = 9), Diffuse slowing (*n* = 9), Bilateral frontotemporal slowing (*n* = 6), Temporal sharp waves (*n* = 2)	Normal (*n* = 12), Unilateral MTL (*n* = 3), Bilateral MTL (*n* = 10)	Mild PL and ⿿Prot (*n* = 5)	N/A	1⿿6 (median = 2.6)	**IT:***n* = 27; steroids PLEX, IVIG and rituximab. **Outcome:** Reduction in seizure frequency >50% (*n* = 14), 20⿿50% (*n* = 12), <20% (*n* = 1)	4 years AEDs: Localized rash (*n* = 8), Erythroderma (*n* = 2), Steven Johnson syndrome (*n* = 2) IT: Steroid induced psychosis (*n* = 1); infection (*n* = 2)
Suleiman et al., 2011	Case control *n* = 4 Female = 3 Double negative (100%)	Encephalopathy, Fever, Behavioural, Seizures	GTC (*n* = 2), SE (*n* = 4), GS (*n* = 2)	Generalized slowing (*n* = 2), Focal slowing (*n* = 2), Epileptic activity (*n* = 1)	Normal (*n* = 2), Left parietal and bifrontal (*n* = 1), Cerebral oedema (*n* = 1)	PL (*n* = 4), ⿿Prot (*n* = 2)	N/A	N/A	**IT:** steroids and IVIG. **Outcome**: Good recovery (*n* = 1), TLE + CI (*n* = 1), TLE + CI + psychiatric (*n* = 1), CI (*n* = 1)	1⿿66 months (median = 15.5)
Quek et al., 2012	Observational *n* = 32 Female = 19 LGI1 (78%) CASPR2 (6%) Double negative (17%)	Seizures, Personality changes, Anxiety/Depression	SPS (*n* = 27), CPS (*n* = 26), GTC (*n* = 17), EPC (*n* = 3)	Epileptiform discharge (*n* = 20), Focal slowing (*n* = 13), Electrographic seizures (*n* = 15), Generalized slowing (*n* = 12)	Normal (*n* = 15), Temporal/extratemporal (*n* = 22)	Normal (*n* = 11), PL (*n* = 5), ⿿Prot (*n* = 17), OCB (*n* = 5)	>2 for 3 weeks⿿12 years (median = 5)	>2 (median = 3)	**IT:***n* = 27; steroids, PLEX. IVIG. **Outcome:** Seizure freedom (*n* = 18), Seizure improvement (*n* = 4), No change (*n* = 5)	3⿿72 months (median = 17)
Shin et al., 2013	Observational *n* = 14 Female = 6 LGI1 (100%)	Seizures, Cognitive dysfunction, Dysautonomia	FBDS (*n* = 10), SE (*n* = 4)	Epileptiform discharge (*n* = 8), Focal slowing (*n* = 2)	Unilateral MTL (*n* = 4), Bilateral MTL (*n* = 5)	Normal (*n* = 10), ⿿Prot (*n* = 3), PL (*n* = 1)	N/A	N/A	**IT:***n* = 14; steroids, PLEX, IVIG, rituximab, tacrolimus, cyclophosphamide, azathioprine. **Outcome**: *n* = 11 (mRS: 0⿿2), *n* = 1 (mRS >2); relapse (*n* = 2)	1⿿24 months (median = 4.5)
Toledano et al., 2014	Observational *n* = 12 Female = 5 LGI1 (92%) CASPR2 (8%)	Seizures	FS (*n* = 6), FBDS (*n* = 4). FS+ (*n* = 10). GTC (*n* = 7)	Normal (*n* = 1), Epileptiform discharges (*n* = 6), Focal slowing (*n* = 2), Excessive beta activity (*n* = 1), Temporal activity (*n* = 1), Extratemporal discharge (*n* = 1)	Normal (*n* = 2), Bilateral MTL (*n* = 3), Right MTL (*n* = 1), Left MTL (*n* = 1), Right MTS (*n* = 1)	Normal (*n* = 5), ⿿Prot (*n* = 6), PL (*n* = 1)	>2 (median = 3)	>1	**IT:***n* = 12; steroids, PLEX, IVIG, mycophenolate mofetil, azathioprine **Outcome:** Seizure freedom (*n* = 10); reduced frequency (*n* = 2)	2⿿86 months With IT: Steroid induced psychosis; aseptic meningitis
Malter et al., 2014	Retrospective *n* = 18 Female = 6 LGI1 (50%) CASPR2 (17%) Double negative (33%)	Amnesia, Confusion, Depression, Seizures, Anxiety	FBDS (*n* = 2)	N/A	Bilateral MTL (*n* = 12) Right MTL (*n* = 2) Left MTL (*n* = 5)	⿿Prot (*n* = 5), PL (*n* = 2)	N/A	>1	**IT:***n* = 18; steroids, mycophenolate mofetil **Outcome:** Seizure free (*n* = 13); Persistent seizures (*n* = 5). No deficit in VM + FM (*n* = 6); Deficit in VM + FM (*n* = 4); Deficit in FM (*n* = 5); Deficit in VM (*n* = 1)	5⿿70 months (median = 26) Steroid-induced liver failure (*n* = 1)
Newey et al., 2014	Observational *n* = 6 Female = 3 VGKC-complex specificity unknown	Seizures, Altered mental state, Depression.	CPS (*n* = 1)	N/A	Unilateral MTL (*n* = 2), Bilateral MTL (*n* = 1)	Normal (*n* = 3), ⿿Prot (*n* = 2), N/A (*n* = 1)	N/A	>2	**IT:***n* = 5 (steroids, PLEX, IVIG). **Outcome:** Complete/partial response (*n* = 3)	N/A
**b. NMDAR-antibody studies**										
Dalmau et al., 2007	Observational *n* = 12 Female = 12	Psychiatric features, Seizures, Movement disorders, Autonomic instability, Reduced level of consciousness	GTC or CPS (*n* = 11)	Diffuse slowing (*n* = 7), Generalized slowing/epileptiform activity (*n* = 3)	Normal (*n* = 3), Bilateral MTL, (*n* = 3), Punctate cortical hyperintensites+/⿿meningeal enhancement (*n* = 5)	PL (*n* = 14), OCB (*n* = 3), ⿿Prot (*n* = 7)	N/A	>2	**IT**: steroids, PLEX, IVIG (*n* = 3); Tumour resection (*n* = 2); IT and tumour resection (*n* = 7). **Outcome**: Full recovery (*n* = 8, MMSE = 28/30), partial recovery (*n* = 2, MMSE = 24/30), Death (*n* = 3)	7 months⿿6 years
Dalmau et al., 2008	Observational *n* = 100 Female = 91	Psychiatric features, Seizures, Movement disorders, Dysautonomia, Hypoventilation	GTC (*n* = 45), CPS (*n* = 10), SE (*n* = 6), FS (*n* = 7), SGTCS (*n* = 8), EPC (*n* = 2), Myoclonus (*n* = 9)	Slow activity (*n* = 71), Epileptic discharge (*n* = 21)	MTL (*n* = 22)	PL (*n* = 91), ⿿Prot (*n* = 32), OCB (*n* = 26)	N/A	N/A	**IT:***n* = 92 (steroids, PLEX, IVIG, cyclophosphamide, rituximab, azathioprine); Tumour resection (*n* = 51). **Outcome:** Full recovery (*n* = 47; **mRS** = 0; MMSE = 29⿿30), Mild stable deficit (*n* = 28 **mRS** = 1⿿2; MMSE = 25 ⿿28) Severe deficit (*n* = 18), Death (*n* = 7)	1⿿194 months (median = 17)
Iizuka et al., 2008	Observational *n* = 4 Female = 4	Psychiatric symptoms, Seizures, Dyskinesia, Hypoventilation	Convulsive seizure (*n* = 2), Tonic (*n* = 1)	Diffuse delta activity no Paroxymal discharge (*n* = 3), Irregular slowing no paroxysmal discharge (*n* = 1)	Normal (*n* = 3), MTL (*n* = 1)	PL (*n* = 4), OCB (*n* = 1)	N/A	>2	**IT:***n* = 2 (steroids, IVIG) **Outcome:** Full recovery (*n* = 2); Gradual recovery over 3⿿4 years (*n* = 2)	4⿿7 years
Niehusmann et al., 2009	Prospective cohort *n* = 5 Female = 5	Psychiatric symptoms, Extratemporal epilepsy Dyskinesia, Dystonia, Hypoventilation, Reduced consciousness	SGTC (*n* = 2), CPS (*n* = 1), SPS + CPS (*n* = 1)	Focal slowing (*n* = 4), Generalized slowing (*n* = 1), Epileptic activity (*n* = 3)	Normal (*n* = 2), White matter (*n* = 1) Cortex (*n* = 1)	PL (*n* = 5), OCB (*n* = 3)	2 months	N/A	**IT**: *n* = 3 (steroids, IVIG); no treatment (*n* = 2). **Outcome:** None relapsed on IT; 2 did not relapse without IT	15⿿36 months (median = 26)
Florance et al., 2009	Observational *n* = 32 Female = 26	Seizures, Behavioural/personality changes, Movement disorders	FS/CPS (*n* = 19), GTC (*n* = 2) SE (*n* = 1), FS/SE (*n* = 1)	Epileptic activity (*n* = 7), Focal or diffuse delta/theta (*n* = 22)	MTL, periventricular and cerebellar (*n* = 5)	PL (*n* = 27), ⿿Prot (*n* = 4), OCB (*n* = 5)	N/A	>2	**IT**: *n* = 30 (Steroids, PLEX. IVIG, rituximab, cyclophosphamide); Tumour resection (*n* = 8); Electroconvulsive therapy (*n* = 2). **Outcome:** Full recovery (*n* = 9), Substantial recovery (*n* = 14), Limited improvement (*n* = 8)	2⿿14.4 months (median = 4.5)
Irani et al., 2010	Observational *n* = 44 Female = 31	Seizures, Confusion, Psychiatric symptoms, Behavioural changes, Movement disorders, Dysautonomia	GTC (*n* = 33) CPS (*n* = 16), SPS (*n* = 12)	Epileptiform discharge (*n* = 22), Generalized slowing in delta or theta range (*n* = 35)	Normal (*n* = 34), Hippocampi (*n* = 4) or white matter (*n* = 6)	PL (*n* = 30), OCB (*n* = 23)	N/A	N/A	**IT**: *n* = 35 (steroids, PLEX, IVIG, cyclophosphamide, rituximab, azathioprine, mycophenolate mofetil). **Outcome:** 75% good recovery (mRS 0 ⿿2)	3.6⿿121 months (median = 16)
Armangue et al., 2013	Observational *n* = 20 Female = 14	Seizures, Psychiatric symptoms, Movement disorders	Seizures (*n* = 12)	Generalized slowing (*n* = 7), Focal slowing (*n* = 3), Generalized slowing/focal activity (*n* = 6). Extreme delta brush (*n* = 1). Epileptiform discharge (*n* = 1)	Temporal lobe (*n* = 6)	PL (*n* = 14), OCB (*n* = 3), ⿿Prot (*n* = 1)	N/A	>2	**IT**: *n* = 20 (steroids, PLEX, IVIG, rituximab, cyclophosphamide). **Outcome**: Full recovery (*n* = 12, PCPC = 1 or 2), Mild disability (*n* = 5, PCPC = 1 or 2), Severe disability (*n* = 2, PCPC = 3 or 4), Death (*n* = 1)	4⿿149 months (median = 17.5)
Titulaer et al., 2013	Observational *n* = 577 Female = 468	Seizures, Psychiatric symptoms, Movement disorders, Behavioural changes, Speech problems, Dysautonomia, Reduction in consciousness	Seizures (*n* = 55)	Slow pattern (*n* = 398), Epileptic features (*n* = 115)	Normal (*n* = 360), Abnormal (*n* = 180), Unknown (*n* = 37)	Normal (*n* = 114), PL (*n* = 402), ⿿Prot (*n* = 93), Unknown (*n* = 45)	N/A	N/A	**IT**: *n* = 472 (steroids, PLEX, IVIG, rituximab, cyclophosphamide, and tumour removal); no treatment (*n* = 29). **Outcome:** Good in 1st 24 months (*n* = 394; mRS = 0⿿2), Good in 2nd 24 months (*n* = 203; mRS = 0⿿2), Poor (*n* = 29; mRS = 3⿿5), One or multiple relapse (*n* = 45)	4⿿186 months (median = 24)
Viaccoz et al., 2014	Observational *n* = 13	Seizures, Amnesia, Dyskinesia, Psychiatry, Cognitive	FS (*n* = 5). GTC (*n* = 3)	Normal (*n* = 2), Slow waves (*n* = 4), SE/seizure (*n* = 6)	Normal (*n* = 6), Hippocampus (*n* = 4), Occipital (*n* = 1), Cerebellar (*n* = 1)	PL (*n* = 10), ⿿Prot/OCB (*n* = 4)	N/A	N/A	**IT:***n* = 12 (steroids, IVIG, cyclophosphamide, rituximab, mycophenolate mofetil). **Outcome:** Favourable (*n* = 10; mRS = 0⿿1), Complete recovery (*n* = 6; mRS = 0⿿1), Death (*n* = 1)	6⿿44 months (median = 14)
Lim et al., 2014	Observational *n* = 40 Female = 15	Seizures, Psychiatric symptoms, Amnesia, Dysautonomia, Movement disorders	Non- convulsive status (*n* = 6)	Epileptic discharge (*n* = 12), Generalized or predominantly frontotemporal slowing (*n* = 10)	MTL (*n* = 3)	PL (*n* = 15), ⿿Prot (*n* = 13)	N/A	N/A	**IT:** IT and/or tumour resection (*n* = 22); No treatment (*n* = 7). **Outcome**: Favourable outcome (*n* = 14; mRS = 0⿿2); Poor outcome (*n* = 7; mRS = 3⿿6)	1⿿12 months (median = 4)
**c. GAD-antibody studies**										
Malter et al., 2010	Observational *n* = 9 Females = 7	Temporal lobe Seizures	TLE (*n* = 9)	N/A	Unilateral hippocampal atrophy (*n* = 3), Bilateral amygdalo-hippocampal signal (*n* = 3)	OCB (*n* = 5), PL (*n* = 2), ⿿Prot (*n* = 2)	N/A	2 (1⿿5)	**IT**: *n* = 9 (steroids, IVIG, cyclophosphamide). **Outcome:** Abnormal VM (*n* = 5); Abnormal FM (*n* = 4). None seizure free	18 months
Haberlandt et al., 2011	Retrospective *n* = 4 Females = 4	Amnesia, Depression, Ataxia, Seizures, Cognitive decline	TLE (*n* = 3). FS (*n* = 1)	N/A	Unilateral MTL (*n* = 2), Bilateral (*n* = 1) MTL	OCB (*n* = 2)	N/A	N/A	**IT**: *n* = 3 (steroids or IVIG). **Outcome**: Memory impairment + TLE (*n* = 2), restitution (*n* = 1), memory impairment + epilepsy (*n* = 1)	13⿿67 months
Lilleker et al., 2014	Observational *n* = 6 Females = 6	Seizures	FS + GTCS (*n* = 5), FS (*n* = 1)	Focal slowing, sharp and slow waves (*n* = 6)	Normal (*n* = 6)	OCB (*n* = 6), ⿿Prot (*n* = 1)	2⿿4 over 9.5 years	>2	**IT**: *n* = 5 (steroids, PLEX, IVIG, azathioprine). Left anterior temporal lobe resection (*n* = 1). **Outcome**: No improvement in seizures (*n* = 4); N/A (*n* = 1)	Optic neuritis (*n* = 1); Transverse myelitis (*n* = 1); Hepatotoxicity (*n* = 1)
**d. GABA**_**B**_**R-antibody studies**										
Lancaster et al., 2010	Observational *n* = 15 Female = 7	Seizures, Amnesia, Confusion, Psychosis	CPS (*n* = 2), FS (*n* = 2), GS(*n* = 5), GTC (*n* = 2), SGTC (*n* = 1). SE (*n* = 3)	Normal (*n* = 1). Temporal lobe seizures, Epileptiform discharge or temporal lobe slowing (*n* = 9). Generalized slowing (*n* = 2)	Normal (*n* = 4), MTL (*n* = 10), Corpus callosum (*n* = 1)	Normal (*n* = 1). PL (*n* = 8), OCB (*n* = 3)	N/A	N/A	**IT**: *n* = 6 (steroid PLEX, IVIG), Tumour resection (*n* = 3), No treatment (*n* = 4). **Outcome:** Good response to IT (*n* = 6) or IT + Tumour resection (*n* = 3), No clinical improvement in untreated patients	3⿿72 months (median = 10)
Boronat et al., 2011	Observational *n* = 10 Female = 1	Seizures, Confusion, Amnesia, Disorientation, Behavioural changes	SE (*n* = 1)	N/A	Normal (*n* = 3). Hippocampus and amygdala (*n* = 7)	PL (*n* = 4)	N/A	N/A	**IT:***n* = 7 (steroids and/or IVIG), Chemotherapy (*n* = 4). **Outcome:** Complete recovery (*n* = 3), Partial response (*n* = 4), No response (*n* = 3)	N/A
Hoftberger et al., 2013	Observational *n* = 20 Female = 8	Seizures, Amnesia, Confusion, Hallucinations, Cerebellar ataxia, Opsoclonus myoclonus	Seizures (*n* = 17), SE (*n* = 1)	Normal (*n* = 5). Epileptic activity with or without generalized slowing (*n* = 7)	Normal (*n* = 7), Unilateral or bilateral MTL (*n* = 9), Pial enhancement (*n* = 1)	PL (*n* = 6), ⿿Prot (*n* = 1), PL/⿿Prot (*n* = 7)	N/A	N/A	**IT**: *n* = 19 (steroids, PLEX, IVIG, rituximab, cyclophosphamide or, mycophenolate mofetil), Chemotherapy (*n* = 4), No treatment (*n* = 3). **Outcome:** Complete recovery (*n* = 7), Partial recovery (*n* = 8)	**0.75**-45 months
Dogan Onugoren et al., 2014	Retrospective *n* = 10 Female = 2	Amnesia, Confusion, Apraxia, Aphasia, Catatonia, Cerebellar dysfunction	GTC (*n* = 9), SE (*n* = 2)	Generalized or focal slowing(*n* = 6), Epileptic activity without slowing (*n* = 2), Epileptic activity with focal or generalized slowing (*n* = 2)	Normal (*n* = 1), Unilateral MTL (*n* = 6), Bilateral MTL (*n* = 3), CLN (*n* = 3)	PL (*n* = 6), OCB (*n* = 7)	N/A	N/A	**IT:***n* = 8 (Steroids, PLEX, IVIG, rituximab, cyclophosphamide, azathioprine), Chemotherapy (*n* = 4), Radiotherapy (*n* = 1). **Outcome:** Full recovery (*n* = 1), Slight recovery (*n* = 1), Decline in cognitive function (*n* = 5), Death (*n* = 3)	2⿿9 months
**e. AMPAR-antibodies studies**										
Lai et al., 2009	Observational *n* = 10 Female = 9	Amnesia, Confusion, Behavioural changes, Disorientation, Seizures	FS (*n* = 1), GTC (*n* = 2)	Normal (*n* = 2), Slow activity (*n* = 2), Sharp waves (*n* = 2), Theta activity (*n* = 1), Epileptic activity (*n* = 1)	Normal (*n* = 1), MTL (*n* = 8), Changes in the anterior septal nuclei (*n* = 2)	OCB (*n* = 3), PL (*n* = 9), ⿿Prot (*n* = 7)	N/A	N/A	**IT:***n* = 9 (steroids, PLEX, IVIG, azathioprine), Tumour resection + IT (*n* = 6). **Outcome**: Good recovery (*n* = 9), Relapses (*n* = 5), Death (*n* = 1)	8⿿50 months (median = 16)
Graus et al., 2010	Retrospective *n* = 4 Female = 4	Confusion, Agitation, Personality changes, Aphasia, No seizures	N/A	Diffuse slowing with occasional sharp waves over the frontal region (*n* = 1), Episodic slow waves in frontal region (*n* = 1)	Normal (*n* = 2)	Normal (*n* = 2)	N/A	N/A	**IT**: *n* = 2 (steroids). **Outcome**: Full recovery (*n* = 2)	N/A
Dogan Onugoren et al., 2014	Retrospective *n* = 3 Female = 1	Amnesia, Psychiatric symptoms, No seizures	N/A	Normal (*n* = 3)	Bilateral MTL (*n* = 3), CLN (*n* = 2)	PL (*n* = 1)	N/A	N/A	**IT**: *n* = 2 (steroids, PLEX, IVIG, azathioprine, rituximab), IT + chemotherapy (*n* = 1). **Outcome:** Good recovery (*n* = 2), Mild recovery (*n* = 1)	5⿿14 months
Hoftberger et al., 2015	Retrospective *n* = 22 Female = 14	Amnesia, Confusion, Insomnia, Seizures	SE (*n* = 1)	Normal (*n* = 4) Epileptiform activity (*n* = 4) Focal activity (*n* = 5) Generalized activity (*n* = 5) Slow activity (*n* = 1) Lateralized periodic slowing (*n* = 1)	Normal (*n* = 4), Unilateral MTL (*n* = 2), Bilateral MTL (*n* = 9)	Normal (*n* = 6), PL (*n* = 11), ⿿Prot (*n* = 10)	N/A	N/A	**IT:***n* = 25 (steroids, PLEX, IVIG, rituximab, cyclophosphamide), Tumour resection (*n* = 7), Chemotherapy (*n* = 8), Radiotherapy (*n* = 6). **Outcome:** Good response (*n* = 5, mRS score, 0⿿1), Partial response (*n* = 10, mRS score, 2⿿3), Poor response (*n* = 6)	5⿿266 weeks (median = 72)
Joubert et al., 2015	Observational *n* = 7 Female = 4	Amnesia, Confusion, Insomnia, Seizures, Cerebellar signs, Tumours	FS (*n* = 1)	Normal (*n* = 4) Focal activity (*n* = 3)	Normal (*n* = 1) Bilateral MTL (*n* = 4) Diffuse T2 hyperintensities (*n* = 2)	Normal (*n* = 1) OCB (*n* = 3), PL (*n* = 5)	1	N/A	**IT:***n* = 7 (steroids, PLEX, IVIG, rituximab, cyclophosphamide, azathioprine). **Outcome**: Good response (*n* = 3, mRS score = 1), Poor response (*n* = 3, mRS score = 3), Death (*n* = 1)	2⿿31 months (median = 12)

**Table 3:** Clinicoradiological characteristics of VGKC-complex, NMDA, GAD and AMPA antibody associated encephalitis. AED = Antiepileptic drugs; AMPAR = Alpha-amino-3-hydroxy-5-methyl-4-isoxazolepropionic acid receptor; CSF = Cerebrospinal fluid; CASPR2 = Contactin-associated protein2; CV2/CRAMP5 = Collapsin response mediator protein; CPS = Complex partial seizure; EEG = Electroencephalogram; FBDS = Faciobrachial dystonic seizures; GTC = Generalized tonic clonic; GABA = Gamma aminobutyric acid; GlyR = Glycine receptor; GluR = Glutamate receptor; GAD = Glutamic acid decarboxylase; LGI1 = Leucine-rich glioma inactivated1; MRI = Magnetic resonance imaging; MTL = Medial temporal lobe; NMDAR = N-methyl-D-aspartate; OCB = Oligoclonal bands; SE = Status epilepticus; SOX1 = Sex determining region Y-box 1; SPS = Stiff Person Syndrome; SCLC = Small cell lung cancer; T1DM = Type 1 diabetes; VGCC = Voltage gated calcium channel.

Characteristic features	LGI1>CASPR2 (VGKC-complex)	NMDAR	GAD	GABA_B_R	AMPAR
Gender	M>F	F>M	F>M	M>F	F>M
Typical age group	>50 years	<40 years	>20 years	> 40 years	> 40 years
Neurological features	Memory loss Confusion Temporal lobe seizures FBDS	Multistage encephalopathy with: Psychiatric symptoms Extratemporal seizures Movement disorders Autonomic instability Coma	Memory loss Temporal lobe seizures Coexisting autoimmune disorders including T1DM, SPS	Memory loss Seizures Confusion	Amnesia Seizures Insomnia Confusion
Psychiatric Features	Psychosis Personality changes Depression Anxiety	Psychosis Behavioural disturbances Delusions Agitation	Depression Anxiety	Psychosis Hallucination Behavioural changes	Psychosis Confabulation Agitation Personality changes
Characteristic seizures	FBDS CPS GTC	GTC SE CPS	GTC CPS	CPS GTC SE Focal motor	GTC CPS
Tumour association	Thymoma SCLC	Ovarian teratoma	SCLC	SCLC	Thymoma SCLC
Target antigen	LGI1 & CASPR2	NR1 subunit	GAD-65	GABA_B_R	GluR1/2
MRI	High signal change in MTL, less commonly basal ganglia	Normal although non-specific signal changes in medial temporal structures	Normal, although increased signal in MTL	Increased signal in MTL	Increased signal in MTL
EEG	Focal or generalized slowing	Extreme delta brush, focal or diffuse delta/theta activity	Focal or generalized slowing	Focal or generalized epileptic activity	Focal epileptic activity
Treatment & outcome	Good response to immunotherapy	Responds slowly to immunotherapy	Poor treatment outcome with immunotherapy and AEDs	Good response to immunotherapy	Relapses are common although there is good response to immunotherapy
