# Supplementary material for: Antibody-associated epilepsies: Clinical features, evidence for immunotherapies and future research questions
Source: Seizure. 2016 Oct;41:26–41. doi: 10.1016/j.seizure.2016.07.002 (PMC5042290; doi:10.1016/j.seizure.2016.07.002)
Supplement: Supplementary file 1 [file mmc1.docx]

**Supplementary Table 1:** Reported treatment data for VGKC complex antibody, NMDAR antibody, GAD antibody and AMPAR antibody associated encephalitis. Details have been included when they were available in the article. IT = Immunotherapy; PLEX = Plasma Exchange; IVIG = Intravenous Immunoglobulins; IVMP = iv methylprednisolone; AEDs = Antiepileptic Drugs; VPA= valproate; LEV = Levetiracetam, LTG=Lamotrigine; LCM = lacosamide; PHT = phenytoin; CBZ = carbamazepine; OXC = oxcarbamazepine; LTG = lamotrigine; PGB = pregabalin; TPM =Topiramate; PHT = phenytoin; ZNS = zonisamide; CLB = clobazam; CLZ = clonazepam; MDZ = Midazolam; PPF = Propofol; DZP = diazepam; TLE = temporal lobe epilepsy; SCLC=small cell lung cancer; N/A= not available.

| **1a. VGKC-complex antibody studies** | | | | | | | | |
| --- | --- | --- | --- | --- | --- | --- | --- | --- |
| **Author** | **Type of study / Number of patients** | **1st line treatment** | **2nd line treatment** | | **Surgery** | | **Other treatment** | **Outcome** |
| Vincent *et al.*, 2004 | Observational  n = 10  female = 1 | IVIG 2g/kg (daily)  Steroids (Prednisolone 100mg (alternate days)  PLEX | N/A | | N/A | | AED (VPA, CLB) | Marked recovery (n =6)  Slight recovery (n= 3)  No recovery (n= 1) |
| Thieben *et al.*, 2004 | Retrospective  n = 7  female = 2 | Steroids (IVMP, 1g daily) | N/A | | N/A | | N/A | Good recovery (n = 3)  Partial recovery (n =3) |
| Mcknight *et al*, 2005 | Case control  n = 16  female = 9 | IVIG  Steroids | N/A | | N/A | | N/A | Good response (n= 5)  No response (n = 1) |
| Lai *et al.*, 2010 | Observational  n = 57 Female=20 | IVIG  Steroids  PLEX | N/A | | N/A | | N/A | Full recovery (n=12)  Mild disability (n=27),  Moderate disability (n=8)  Relapse (n=6) |
| Irani *et al.*, 2011 | Observational  n =29  Female = 10 | IVIG  Steroids  PLEX | Rituximab | | N/A | | N/A | Reduction in seizure frequency  >50% (n =14),  20-50% (n= 12)  <20% (n= 1) |
| Suleiman *et al.*, 2011 | Case control  n = 4  Female = 3 | IVIG  Steroids | N/A | | N/A | | N/A | Good recovery (n=1)  TLE + cognitive impairments (n=2).  TLE + cognitive impairments + psychiatric abnormalities (n=1)  Cognitive impairment (n=1) |
| Quek *et al.*, 2012 | Observational  n = 32  Female = 19 | IVIG  Steriods  PLEX | Cyclophosphamide  Mycophenolate mofetil  Rituximab  Azathioprine  Methotrexate | | Tumour resection | | AED (LEV, LTG) | Seizure freedom (n=18)  Seizure improvement (n=4),  No change (n=5) |
| Shin *et al*, 2013 | Observational n=14 Female=6 | IVIG (400mg/kg x5 days),  Steroids (IVMP, 500mg -1g x5days),  PLEX | Rituximab, tacrolimus, cyclophosphamide, azathioprine. | | N/A | | N/A | No relapse(n=11)  Relapse (n=2)  N/A (n=1) |
| Toledano *et al,* 2014 | Observational n=12  Female = 5 | IVIG(0.4g/kg/d for 3-5days)  Steroids (IVMP, 500mg-1g for 3-5days)  PLEX | Azathioprine Mycophenolate mofetil | |  | | AED(LCM, PHT, LEV, CBZ, OXC, LTG, PGB ) | Seizure freedom (n=10)  Reduced seizure frequency (n=2) |
| Newey *et al.,* 2014 | Observational  n=6  Female =3 | IVIG (0.4g/kg/d for 3-5days)  Steroids ( IVMP, 1g daily for 3-5days)  PLEX (3days per week for 2weeks) | N/A | | N/A | | AED (TPM,PHT, ZNS, LEV, LTG, VPA) | Complete/partial response to IT (n=3) |
| **1b. NMDAR-antibody studies** | | | | | | | | |
| **Author** | **Type of study / Number of patients** | **1st line treatment** | **2nd line treatment** | | **Surgery** | | **Other treatment** | **Outcome** |
| Dalmau *et al.*, 2007 | Observational  n=12 Female = 12 | IVIG  Steroids  PLEX | Cyclophosphamide | | Tumour resection (ovarian teratoma) | | Chemotherapy | IT or tumor resection (full recovery (n=2), partial recovery (n=1), death from neurological progression (n=2)  IT and tumour resection (Full recovery (n=5), partial recovery (n=1), death after mild improvement (n=1) |
| Dalmau *et al.*, 2008 | Observational  n=100  Female = 91 | IVIG  Steroids  PLEX | Cyclophosphamide,  Rituximab,  Azathioprine. | | Tumour resection (ovarian teratoma) | | Electroconvulsive therapy  Chemotherapy. | Full recovery (n=47), mild stable deficit (n=28), severe deficit (n=18), death (n=7)  Patient’s with tumours resected at 4 months made better recovery than those with tumours resected >4 months |
| Iizuka *et al.*,  2008 | Observational  n=4  Female = 4 | IVIG  Steroids | N/A | | N/A | | AED (PHT, CLB ZNS, CLZ, VPA)  Acyclovir  MDZ, PPF, phenobarbital,  Pentobarbital,  Quetiapine, Trihexyphenidyl  Haloperidol | Full recovery (n=2)  Gradual recovery over  3 – 4 years (n=2). |
| Niehusmann  *et al.*, 2009 | Prospective cohort  n=5  Female=5 | IVIG  Steroids  Prednisolone | N/A | | N/A | | N/A | 3 patients recovered after IT. None relapsed while undergoing IT. |
| Florance *et al.*, 2009 | Observational  n=32  Female = 26 | IVIG  Steroids  PLEX | Cyclophosphamide Rituximab | | Tumour resection  (ovarian teratoma) | | Electroconvulsive therapy | Full recovery (n=9)  Substantial recovery (n=14)  Limited improvement (n=8) |
| Irani *et al.*, 2010 | Observational  n=44  Female = 31 | IVIG  Steroids  PLEX | Cyclophosphamide,  Rituximab  Azathioprine  Mycophenolate mofetil | | Tumour resection (ovarian teratoma) | | N/A | 23% good recovery |
| Armangue *et al.* 2013 | Observational  n=20  Female = 14 | IVIG  Steroids  PLEX | Cyclophosphamide  Rituximab,  Mycophenolate mofetil. | | Tumour resection (ovarian teratoma) | | AED, Terabenazine, piracetam. | Full recovery (n=12)  Mild disability (n=5)  Moderate disability (n=2),  Coma (n=1) |
| Titulaer *et al.*, 2013 | Observational  n=577  female = 468 | IVIG  Steroids  PLEX | Cyclophosphamide  Rituximab  Azathioprine  Tacrolimus or Methotrexate  Mycophenolate mofetil | | Tumour resection (ovarian teratoma) | | **N/A** | Good in 1st 24 months (n=394)  Good in 2nd 24 months (n=204)  One or multiple relapse (n=45)  Death (n=30) |
| Lim *et al,* 2014 | Observational n=40  female = 15 | IVIG  Steroids  PLEX | Cyclophosphamide  Rituximab. | | Tumour resection (ovarian teratoma, colon cancer) | | N/A | Favourable outcome (n=14)  Poor outcome (n=7)  Loss of consciousness |
| **1c. GAD-antibody studies** | | | | | | | | |
| **Author** | **Type of study / Number of patients** | **1st line treatment** | **2nd line treatment** | | **Surgery** | | **Other treatment** | **Outcome** |
| Malter *et al.*, 2010 | Observational  N=9  Female =7 | Steroids (IVMP, 500-1000mg/d, 3-5days)  IVIG | cyclophosphamide | | N/A | | AED | None became seizure free |
| Haberlandt *et al.*, 2011 | Retrospective  n =4  Females=4 | IVIG  Steroids | N/A | | N/A | | N/A | Memory impairment + TLE (n=2), restitution (n=1), memory impairment + epilepsy (n=1). |
| Lilleker *et al.*, 2014 | Observational  n=6  Females=6 | IVIG  Steroids  PLEX | Azathioprine | | Left anterior temporal lobe resection. | | AED (CBZ, LTG, ZNS , LEV, PGB, OXC, CLB) | No improvement in seizures after IT (n=4)  N/A (n=1)  Post operatively, patient was seizure free for 10months followed by occasional focal seizures. |
| **1d. GABA_B_R-antibody studies** | | | | | | | | |
| **Author** | **Type of study / Number of patients** | **1st line treatment** | **2nd line treatment** | | **Surgery** | | **Other treatment** | **Outcome** |
| Lancaster *et al.*, 2010 | Observational  n=15  Female = 7 | IVIG  Steroids  PLEX | Mycophenylate mofetil | | Tumour resection.  (lobectomy for SCLC | | Chemotherapy with cisplatin and etoposide.  AED (LEV, VPA, PHT) | Good response to IT (n=6)  IT + Tumour resection (n=3)  No clinical improvements in patients who did not receive treatment (n=4) |
| Boronat *et al.*, 2011 | Observational  n=10  Female=1 | IVIG  Steroids | N/A | | N/A | | Chemotherapy.  AED | Good recovery (n=3)  Partial response (n=4)  No response (n=3) |
| Hoftberger *et al*, 2013 | Observational  n= 20  Female = 8 | IVIG  Steroids  PLEX | Cyclophosphamide  Rituximab  Mycophenolate mofetil. | | N/A | | Chemotherapy  Radiation therapy | Complete recovery (n=7)  Partial recovery (n=8) |
| [Dogan Onugoren](http://ezproxy-prd.bodleian.ox.ac.uk:2828/search?author1=M+Dogan+Onugoren&sortspec=date&submit=Submit) *et al*, 2014 | Retrospective.  n=10  Female=2 | IVIG  Steroids  PLEX | Rituximab  Cyclophosphamide  Azathioprine | | N/A | | Chemotherapy  Radiotherapy | Complete recovery (n=1).  Slight recovery (n=1)  Decline in cognitive function (n=5),  Death(n=3) |
| Petit pedro *et al.*, 2014 | Observational  n=6  Female=1 | IVIG  Steroids  PLEX | Rituximab  Cyclophosphamide | | Decompressive posterior craniectomy | | AED(LEV,TPM, LCM, PHT, CLB, VPA)  DZP  Barbiturates | Full/partial recovery (n =3)  Substantial recovery (n= 1)  Death (n=2) |
| **1e. AMPAR-antibody studies** | | | | | | | | |
| **Author** | **Type of study / Number of patients** | **1st line treatment** | | **2nd line treatment** | **Surgery** | **Other treatment** | | **Outcome** |
| Lai *et al*, 2009 | Observational  n=10  Female = 9 | IVIG  Steroids  PLEX | Azathioprine | | Tumour resection (thymic carcinoma, malignant thymoma) | | Chemotherapy  Radiation therapy | Good recovery (n=9)  Relapse (n=5)  Death (n=1) |
| Graus *et al* 2010 | Retrospective.  n= 4  Female=4 | Steroids | N/A | | N/A | | Chemotherapy | Complete recovery(n=2) |
| [Dogan Onugoren](http://ezproxy-prd.bodleian.ox.ac.uk:2828/search?author1=M+Dogan+Onugoren&sortspec=date&submit=Submit) *et al* 2014 | Retrospective  n=3  Female=1 | IVIG  Steroids  PLEX | Rituximab  Azathioprine  Mycophenolate mofetil | | Tumour resection (ovarian cancer) | | Chemotherapy | Good recovery (n=2)  Mild recovery (n=1) |

**Supplementary Table 2. Studies with MRI findings involving multiple brain regions.**

| **Studies** | **MRI findings.** |
| --- | --- |
| Shin et al 2013 | Multiple lesions in bilateral basal ganglia, thalamus, white mater and central pons (n=1). |
| Toledano et al 2014 | Left MTL + bilateral MTS (n=2), Left MTL + left caudate and putamen+ left MTS (n=1), right frontal lobe (n=1). |
| Newey et al 2014 | Global atrophy, laminar necrosis in left parietal lobe (n=1), Bilateral basal ganglia and hippocampal (n=1), Restiform bodies (n=1), Hypothalamus (n=3). |
| Dalmau et al 2008 | Cerebellum (n=6), Brainstem (n=6), Corpus callosum (n=4), periventricular (n=1), multifocal white matter (n=1), contrast enhancement in meninges, cortex and basal ganglia (n=14), Cerebral cortex (n=17), Basal ganglia (n=5), Hypothalamus (n=2). |
| Florence et al 2009 | Transient leptomeningeal enhancement (n=2), transient cortical enhancement + sulci abnormalities (n=1), mild mesial temporal atrophy (n=1), multifocal parenchyma enhancement (n=1). |
| Armangue et al 2013 | Minimal changes in insular region (n=1), Cystic lesions left temporal lobe with perilesional gliosis (n=1), Transient brain atrophy (n=1). |
| Lim et al 2014 | Cerebral cortex (n=7), Basal ganglia (n=1), periventricular or subcortical white mater (n=3), Contrast enhancement in cortex  and meninges (n=3). |
